# Keap1-Nrf2 signaling activation by Bardoxolone-methyl ameliorates high glucose-induced oxidative injury in human umbilical vein endothelial cells

**DOI:** 10.18632/aging.103263

**Published:** 2020-06-02

**Authors:** Jing-Lei Yang, Meng-Yue Sun, Qi Yuan, Shan Tang, Mei-Juan Dong, Ri-Dong Zhang, Yuan-Yuan Liu, Li Mao

**Affiliations:** 1 Department of Endocrinology, The Affiliated Huai’an People’s Hospital of Nanjing Medical University, Huai’an, China

**Keywords:** bardoxolone-methyl, high glucose, HUVECs, Keap1-Nrf2 cascade, oxidative injury

## Abstract

In cultured human umbilical vein endothelial cells (HUVECs) high glucose (HG) stimulation will lead to significant cell death. Bardoxolone-methyl (BARD) is a NF-E2 p45-related factor 2 (Nrf2) agonist. In this study we show that BARD, at only nM concentrations, activated Nrf2 signaling in HUVECs. BARD induced Keap1-Nrf2 disassociation, Nrf2 protein stabilization and nuclear translocation, increasing expression of antioxidant response element (ARE) genes. BARD pretreatment in HUVECs inhibited HG-induced reactive oxygen species production, oxidative injury and cell apoptosis. Nrf2 shRNA or knockout (using a CRISPR/Cas9 construct) reversed BARD-induced cytoprotection in HG-stimulated HUVECs. Conversely, forced activation of Nrf2 cascade by Keap1 shRNA mimicked BARD’s activity and protected HUVECs from HG. Importantly, BARD failed to offer further cytoprotection against HG in the Keap1-silened HUVECs. Taken together, Keap1-Nrf2 cascade activation by BARD protects HUVECs from HG-induced oxidative injury.

## INTRODUCTION

Vascular endothelial cell injury is a primary medical issue in the pathogenesis and progression of cardiovascular complications in diabetes mellitus patients [1, 2]. Sustained high glucose (HG) exposure to vascular endothelial cells will induce robust reactive oxygen species (ROS) production and oxidative injury. Subsequently, it will induce calcium overload, lipid peroxidation, as well as profound protein and DNA damage. These events together will induce cell death and apoptosis [3–7]. Human umbilical vein endothelial cells (HUVECs) were cultured with HG-containing medium, mimicking diabetes mellitus-induced pathogenesis in vascular endothelial cells [8–13].

NF-E2 p45-related factor 2 (Nrf2) is a mater transcriptional factor suppressing cellular oxidative injury and other stresses [14–18]. Once activated, the transcription factor will depart from its suppressor protein Keap1. The Nrf2 protein will be then stabilized and translocate to cell nuclei. At last it will bind to antioxidant response element (ARE), essential for the basal and inducible expression of many different genes. These genes encode detoxification enzymes, antioxidant proteins and many other cytoprotective proteins [14–18].

Studies have demonstrated that forced activation of Keap1-Nrf2 cascade can efficiently protect vascular endothelial cells from HG and other oxidative injury. Tang et al., demonstrated that 4-octyl itaconate (OI) protected HUVECs from HG through activation of Nrf2 signaling cascade [8]. A study by Yang et al., showed that phloretin activated AMPK (AMP-activated protein kinase)-Nrf2 cascade to alleviate endothelial cell injury by palmitic acid [19]. Zeng et al., reported that a long non-coding RNA MALAT1 activated Nrf2 cascade to protect HUVECs from hydrogen peroxide [20]. Therefore, Nrf2 activation should be a valuable strategy to protect endothelial cells from oxidative injury.

Bardoxolone-methyl (BARD) is a synthetic triterpenoid [21–25]. BARD has displayed antioxidant, anti-inflammatory, anti-proliferative and anti-fibrotic activities under experimental and clinical settings [21–25]. BARD is an extremely efficient activator of Nrf2. It directly binds to Keap1, thus preventing it from interaction with Skp1-Cul1-Rbx1/Roc1 complex [22, 25]. Therefore, BARD will induce release of activate Nrf2, causing Nrf2 protein stabilization and nuclear translocation [26, 27]. It will eventually promote expression of ARE-dependent genes. These genes include *heme oxygenase-1* (*HO1*), *NAD(P)H quinone oxidoreductase 1* (*NQO1*) and *γ-glutamyl cysteine ligase catalytic subunit* (*GCLC)* [18]. The results of the present study will show that BARD activates Nrf2 signaling to protect HUVECs from HG-induced oxidative injury.

## RESULTS

### BARD robustly activates Nrf2 signaling cascade in HUVECs

**Figure 1:**
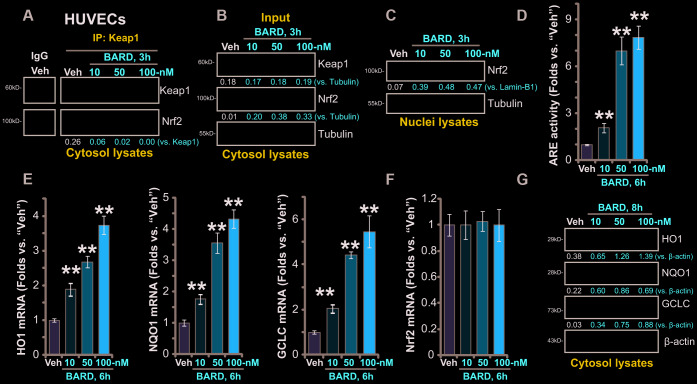
**BARD robustly activates Nrf2 signaling cascade in HUVECs.** Human umbilical vein endothelial cells (HUVECs) were treated with Bardoxolone Methyl (BARD, at 10-100 nM) and cultured for applied time periods, Nrf2-Keap1 binding was tested by a co-immunoprecipitation assay (**A**); Expression of listed protein in cytosol fraction lysates (**B**, **G**) and nuclear fraction lysates (**C**) was tested by Western blotting, with expression of listed Nrf2 pathway mRNAs examined by qPCR (**E**, **F**); The relatively ARE (antioxidant response element) activity was also tested (**D**). Expression of the listed proteins was quantified, normalizing to the indicated loading control protein. (**A**–**C**, **G**) Error bars stand for mean ± standard deviation (SD, n=5). “Veh” stands for vehicle control (same for all Figures). ** *p*<0.01 *vs.* “Veh” (**D**, **E**) Each experiment was repeated five times to insure the consistency of experimental results.

BARD can induce Nrf2 signaling cascade activation by releasing Nrf2 from Keap1 [21, 22]. A co-immunoprecipitation (Co-IP) assay was carried out in cultured HUVECs. Results, in Figure 1A, demonstrated that the cytosol Keap1-Nrf2 association was disrupted with treatment of BARD (10-100 nM) for 3h. The input control results demonstrated that Nrf2 protein levels were elevated in BARD-treated HUVECs (Figure 1B), where Keap1 levels were unchanged (Figure 1B). By testing the nuclear fraction proteins, we found that the Nrf2 protein was enriched in the nuclei of BARD (10-100 nM)-treated HUVECs, with significant increase of ARE activity (Figure 1D). Based on the results we propose that BARD treatment disrupted Nrf2-Keap1 binding, causing cytosol Nrf2 protein stabilization and nuclear translocation, thus increasing ARE activity in HUVECs.

Further results show that mRNA expression of Nrf2-ARE-dependent genes, including *HO1*, *NQO1* and *GCLC*, was significantly increased following BARD (10-100 nM) treatment in HUVECs (Figure 1E). Expression of *Nrf2 mRNA* was, however, unchanged (Figure 1F). Protein levels of HO1, NQO1 and GCLC were augmented as well in BARD-treated HUVECs (Figure 1G). Therefore, BARD efficiently (at nM concentrations) activated Nrf2 signaling cascade in HUVECs. Since 50 nM BARD induced robust Nrf2 cascade activation, this concentration was chosen for the following studies.

### BARD inhibits high glucose-induced oxidative injury in HUVECs

**Figure 2:**
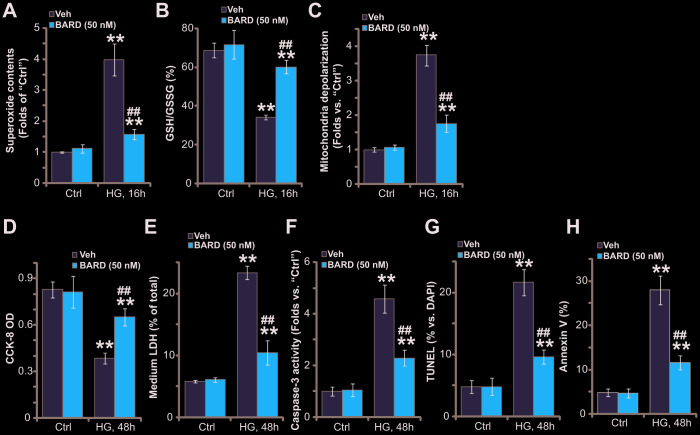
**BARD inhibits high glucose-induced oxidative injury in HUVECs.** HUVECs were pretreated with Bardoxolone Methyl (BARD, at 50 nM) for 1h, followed by HG stimulation and cultured for applied time periods, the cellular superoxide contents (**A**), the GSH/GSSH ratio (**B**) and mitochondrial depolarization (JC-1 green intensity, **C**) were tested; Cell viability and death were tested by CCK-8 (**D**) and medium LDH release (**E**) assays, respectively, with cell apoptosis analyzed by caspase-3 activity (**F**), nuclear TUNEL staining (**G**) and Annexin V-FACS (**H**) assays. For TUNEL staining assays, at least 500 nuclei in five random views (1×200 magnification) for each condition were included to calculate the TUNEL/DAPI ratio (same for all Figures). Error bars stand for mean ± standard deviation (SD, n=5). “Ctrl” stands for cells-cultured in the normal glucose medium (same for all Figures). ** *p*<0.01 *vs.* “Ctrl” treatment. ^##^*p*<0.01. *vs.* HG only treatment (no BARD pretreatment). Each experiment was repeated five times to insure the consistency of experimental results.

High glucose (HG) treatment in HUVECs can induce robust oxidative injury, responsible for following cell death and apoptosis [8, 28–31]. Contrarily, antioxidant agents or genetic strategies suppressing oxidative injury can protect HUVECs from HG [8, 28, 31]. We here also found that HG induced potent oxidative stress in HUVECs, leading to superoxide accumulation (Figure 2A), GSH reduction (a GSH/GSSG ratio decrease, Figure 2B) and significant mitochondrial depolarization (green JC-1 monomers accumulation, Figure 2C), which were largely attenuated by pretreatment of BARD (50 nM, 1h) (Figure 2A–2C).

Further studies demonstrated that HG stimulation for 48h led to significant viability (CCK-8 OD) reduction (Figure 2D) and cell death (medium LDH release, Figure 2E). Importantly, BARD pretreatment potently attenuated HG-induced cytotoxicity in HUVECs (Figure 2D, 2E). Additionally, significant apoptosis activation was detected in HG-treated HUVECs, which was reflected in the increase of caspase-3activity (Figure 2F), nuclear TUNEL staining (Figure 2G) and Annexin V ratio (Figure 2H). BARD pretreatment largely attenuated HG-induced apoptosis in HUVECs as well (Figure 2F, 2G). Collectively, BARD pretreatment potently inhibited HG-induced oxidative injury in HUVECs.

### Nrf2 silencing or knockout blocks BARD-induced cytoprotection in HG-stimulated HUVECs

**Figure 3:**
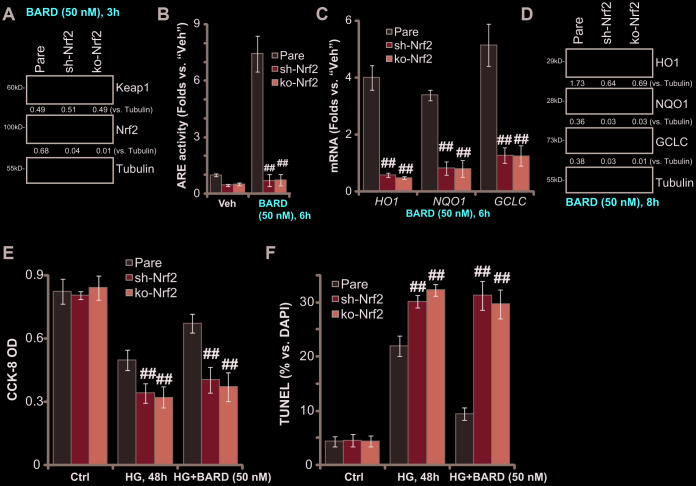
**Nrf2 silencing or knockout blocks BARD-induced cytoprotection in HG-stimulated HUVECs.** The stable HUVECs with Nrf2 shRNA lentiviral particles (“sh-Nrf2”) or the lenti-CRISPR-GFP-Nrf2 knockout (KO) construct (“ko-Nrf2”), as well as the parental control cells (“Pare”), were treated with Bardoxolone Methyl (BARD, at 50 nM) for applied time periods, expression of listed genes was tested by qPCR and Western blotting analyses (**A**, **C**, **D**); The relative ARE activity was examined as well (**B**); Alternatively, cells were pretreated with BARD (50 nM) for 1h, followed by HG stimulation and cultured for 48h, cell viability (CCK-8 assay, **E**) and apoptosis (nuclear TUNEL staining assay, **F**) were tested. Expression of the listed proteins was quantified, normalizing to the indicated loading control protein (**A**, **D**). Error bars stand for mean ± standard deviation (SD, n=5). ^##^*p*<0.01. vs. “Pare” cells. Each experiment was repeated five times to insure the consistency of experimental results.

To test whether Nrf2 signaling activation was required for BARD-induced cytoprotection in HG-stimulated HUVECs, a shRNA strategy was applied to silence Nrf2 in HUVECs, and stable cells (“sh-Nrf2”) established with puromycin selection. Furthermore, the stable HUVECs with the lenti-CRISPR-GFP-Nrf2 knockout (KO) construct (“ko-Nrf2”, provided by Dr. Xu [8]) were utilized. As shown BARD-induced Nrf2 protein stabilization was completely abolished in sh-Nrf2-HUVECs and ko-Nrf2-HUVECs (Figure 3A). The increase of ARE activity in response to BARD was also reversed with Nrf2 silencing or KO (Figure 3B). Additional experimental results demonstrated that BARD-induced mRNA and protein expression of ARE-dependent genes, *HO1*, *NQO1* and *GCLC*, was completely blocked with Nrf2 shRNA or KO (Figure 3C, 3D). In sh-Nrf2-HUVECs and ko-Nrf2-HUVECs HG-induced viability (CCK-8 OD) reduction (Figure 3E) and apoptosis (nuclear TUNEL ratio, Figure 3F) were intensified (*vs.* parental control cells/“Pare”). Importantly, BARD was completely ineffective on HG-induced cytotoxicity in sh-Nrf2-HUVECs and ko-Nrf2 HUVECs (Figure 3E, 3F). These results show that Nrf2 silencing or KO abolished BARD-induced cytoprotection in HG-stimulated HUVECs, suggesting that Nrf2 cascade activation is required for BARD-induced activity in HUVECs.

### Keap1 silencing mimics BARD-induced cytoprotection in HG-stimulated HUVECs

**Figure 4:**
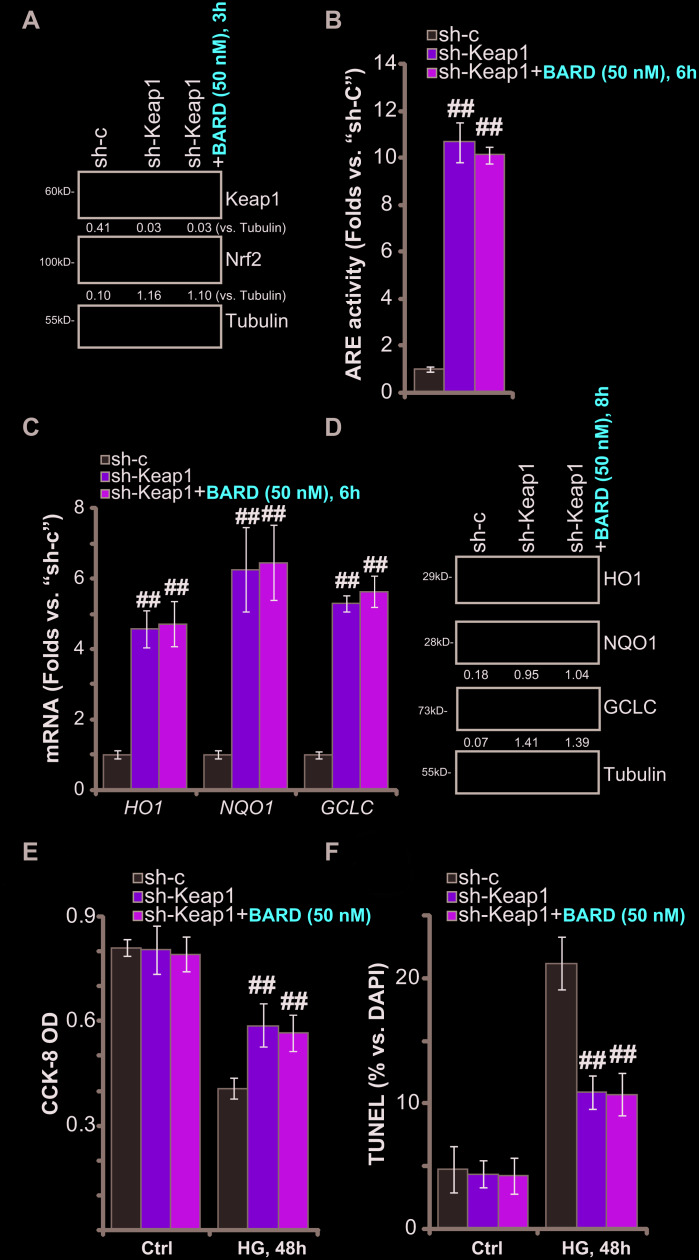
**Keap1 silencing mimics BARD-induced cytoprotection in HG-stimulated HUVECs.** The stable HUVECs with Keap1 shRNA lentiviral particles (“sh-Keap1”) were treated with or without Bardoxolone Methyl (BARD, at 50 nM) for applied time periods, control cells were transduced with the scramble control shRNA (“sh-C”), expression of listed genes was tested by qPCR and Western blotting analyses (**A**, **C**, **D**), with the relative ARE activity examined as well (**B**); Alternatively, cells were pretreated with BARD (50 nM) for 1h, followed by HG stimulation and cultured for 48h, cell viability (CCK-8 assay, **E**) and apoptosis (nuclear TUNEL staining assay, **F**) were tested. Expression of the listed proteins was quantified, normalizing to the indicated loading control protein (A and D). Error bars stand for mean ± standard deviation (SD, n=5). ^##^*p*<0.01. vs. “sh-C” cells. Each experiment was repeated five times to insure the consistency of experimental results.

BARD activates Nrf2 signaling cascade through disrupting Nrf2-Keap1 association and inhibiting Nrf2 protein degradation [21–25]. We therefore proposed that Nrf2 activation by Keap1 silencing should mimic BARD-induced cytoprotection in HG-stimulated HUVECs. Therefore, Keap1 shRNA lentiviral particles were added to cultured HUVECs, resulting in over 90% silencing of the Keap1 protein in the stable cells (“sh-Keap1” cells) (Figure 4A). As shown Keap1 shRNA led to robust Nrf2 protein stabilization (Figure 4A) and significant increase of the ARE activity (Figure 4B) in HUVECs. Furthermore, mRNA and protein expression of *HO1*, *NQO1* and *GCLC* was augmented in Keap1-silenced HUVECs (Figure 4C, 4D). Thus, shRNA-mediated silencing of Keap1 mimicked BARD-induced activity and activated Nrf2 cascade in HUVECs.

Importantly, in the Keap1-silenced HUVECs, treatment with BARD failed to further increase Nrf2 protein levels (Figure 4A), ARE activity (Figure 4B) or the expression of ARE-dependent genes (*HO1*, *NQO1* and *GCLC*, Figure 4C, 4D). Therefore, BARD was ineffective on Nrf2 signaling in HUVECs with Keap1 silencing. Functional studies demonstrated that sh-Keap1-HUVECs were protected from HG, presenting with significantly decreased viability (CCK-8 OD) reduction (Figure 4E) and apoptosis activation (Figure 4F), when compared to the control HUVECs with scramble non-sense shRNA lentivirus (“sh-C”). Significantly, in the sh-Keap1-HUVECs treatment with BARD failed to offer further cytoprotection against HG-induced cell death and apoptosis (Figure 4E, 4F). These results further suggest that activation of Keap1-Nrf2 cascade mediated BARD-induced cytoprotection in HG-stimulated HUVECs.

## DISCUSSION

Under the resting conditions, Nrf2 binds to its suppressor protein Keap1. It is distributed in an inactive state and eventually degraded in the cytosol. Following activation, the Nrf2 protein will disassociate with Keap1, causing Nrf2 cytoplasmic entry into the nuclei and binding to antioxidant genes. This will promote transcription and activate cellular antioxidant functions [17, 18, 32]. The emerging studies have reported that BARD could activate Nrf2 signaling and ameliorate oxidative stress through disrupting Keap1-Nrf2 association and induction of antioxidant genes [22, 33, 34].

In the present study we show that BARD induced robust Nrf2 signaling activation in HUVECs. Following BARD treatment Nrf2 departed from KEAP1 in cytosol, causing Nrf2 protein stabilization. The active Nrf2 protein then translocated to cell nuclei in HUVECs, promoting transcription of ARE genes, including *HO1*, *NQO1* and *GCLC*. Importantly, HG-induced ROS production, oxidative injury and cell death/apoptosis were largely attenuated by BARD pretreatment in HUVECs. Thus, BARD activated Nrf2 cascade and ameliorated HG-induced oxidative injury in HUVECs.

Our results implied that activation of Keap1-Nrf2 signaling is required for BARD-induced cytoprotection against HG in HUVECs. Nrf2 silencing or KO completely blocked BARD-induced increases of ARE activity and expression of ARE genes (*HO1*, *NQO1* and *GCLC*). Furthermore, BARD-induced cytoprotection against HG was completely reversed with Nrf2 silencing or KO in HUVECs. Further studies found that forced Nrf2 cascade activation by Keap1 shRNA mimicked BARD’s activity and protected HUVECs from HG-induced cell death/apoptosis. Importantly, BARD failed to offer further cytoprotection against HG in the Keap1-silened HUVECs. Therefore, Keap1 depletion not only mimicked, but also nullified, BARD-induced cytoprotection against HG in HUVECs. These results confirm that in HUVECs BARD-induced cytoprotection against HG requires Keap1-Nrf2 cascade activation.

As compared to other known Nrf2 activators, one significant and obvious advantage of this compound is that it directly acts on Keap1, causing fast and sustained Keap1-Nrf2 disassociation and profound Nrf2 cascade activation [22, 23, 34]. Therefore, BARD is an extremely efficient and promising Nrf2 activator.

Phase II clinical trials in chronic kidney diseases (CDKs) patients have demonstrated a long-term increment in glomerular filtration by BARD administration [35]. However, a phase III clinical trial evaluating patients with type 2 diabetes mellitus and stage 4 CDKs revealed that BARD failed to reduce the risk of end-stage renal disease (ESRD) or death from cardiovascular causes [36]. The trial was terminated due to a high rate of heart-related adverse events in patients, including nonfatal myocardial infarction, nonfatal stroke, heart failure, or death from cardiovascular causes [36]. Interestingly, a new phase II clinical trial is recruiting rare CKD patients to better define the safety and efficacy profiles of BARD [37]. Furthermore, a phase II clinical trial is ongoing to evaluate BARD in pulmonary arterial hypertension patients [37].

It should be noted that the current results from *in vitro* studies shall not be directly translated to humans. A single concentration of HG stimulation in cultured HUVECs is also not equal to vascular injury in the diabetes mellitus patients. The efficacy and safety of BARD will need further *in vivo* characterizations.

## CONCLUSIONS

Taken together, we conclude that Keap1-Nrf2 cascade activation by BARD protects HUVECs from HG-induced oxidative injury.

## MATERIALS AND METHODS

### Materials and reagents

BARD, CCK-8 dye and puromycin were provided by Sigma-Aldrich (St. Louis, MO). Antibodies utilized in this study were provided by Santa Cruz Biotechnology (Santa Cruz, CA) and Abcam (Shanghai, China). The cell culture reagents, fetal bovine serum (FBS) and others, were obtained from Hyclone Co. (Logan, UT). TRIzol along with other PCR agents and Lipofectamine 2000 were provided by Thermo-Fisher Invitrogen (Shanghai, China). All the primers and sequences were synthesized by Shanghai Genechem Co. (Shanghai, China).

### Culture of HUVECs

HUVECs were provided by Dr. Jiang [38] at Nanjing Medical University, cultured in the described medium [8, 39]. For high glucose (HG) stimulation, HUVECs were cultured in the basal medium plus 50 mM glucose. Cells were further cultured for indicated time periods, before further biological analyses were performed. The protocols of this study were approved by the Ethics Committee of Nanjing Medical University.

### Quantitative real-time reverse transcription PCR assay (qPCR)

Following the applied treatment, the total cellular RNA of HUVECs was extracted by TRIzol, that was reversely transcribed using a ReverTra Ace qPCR RT kit (Toyobo). qPCR was performed under an ABI Prism 7900 HT System (Applied Biosystems, Foster City, CA). mRNA primers for *Nrf2*, *HO1*, *NQO1*, and *GCLC* were listed in Table 1. The product melting temperature was always calculated [40]. Quantization of the listed mRNAs was carried out through a 2^−∆∆*C*t^ method, with *GAPDH* tested as the reference gene.

**Table 1:** Primers utilized in this study.

**Gene name**	**Forward primer (5’-3’)**	**Reverse primer (5’-3’)**
*NQO1* (NM_000903)	CATTCTGAAAGGCTGGTTTG	GGCTGCTTGGAGCAAAATAC
*HO1* (NM_002133)	GCTACCTGGGTGACCTGTCT	GGGCAGAATCTTGCACTTTG
*Nrf2* (NM_006164)	TGAGCATGCTTCCCATGAT	CTTCTCTAGCCGCTCTGTGG
*GAPDH* (NM_002046)	CGGAGTCAACGGATTTGGTCGTAT	AGCCTTCTCCATGGTGGTGAAGAC
GCLC (NM_001498)	GGAAGTGGATGTGGACACCAGA	GCTTGTAGTCAGGATGGTTTGCG

### ARE reporter assay

HUVECs were initially seeded onto a six-well plate (at 2 × 10^5^ cells/well), and transfected with an ARE-inducible firefly luciferase vector (provided by Dr. Jiang at Nanjing Medical University [41]). The transfected cells were treated with BARD and cell lysates subjected to tests of the luciferase activity using a luminescence machine.

### Western blotting

HUVECs with applied treatments were incubated with the described lysis buffer [42]. For each treatment, 40 μg of protein lysates were separated in a denaturing 10% SDS-PAGE gel, thereafter transferred onto a PVDF blot (Millipore, Shanghai, China). After blocking, the blot was incubated with applied primary antibody and corresponding secondary antibody at appropriate concentrations. An enhanced chemiluminescence (ECL) kit (Amersham) was utilized to test antibody-antigen binding based on the molecular weight of the targeted protein. An ImageJ software (National Institutes of Health, Bethesda, MD) was utilized for data quantification [43, 44]. The separation of nuclear fraction lysates through a kit from Sigma was described previously [45].

### Co-Immunoprecipitation (Co-IP)

The detailed protocols for Co-IP were described in elsewhere [44]. Briefly, following the applied BARD treatment, 1 mg of total cell lysates from HUVECs were pre-cleared by adding the protein A/G Sepharose (“Beads”). Afterwards, an anti-Keap1 antibody (15 μL for each treatment, Sana Cruz Biotech, Sana Cruz) was added to the pre-cleared lysates overnight. Thereafter, the protein A/G Sepharose were added back to the lysates for 3h. The Keap1-immunoprecipitated proteins were subjected to Western blotting analyses.

### Cell viability

At the density of 30, 000 cells/cm^2^ HUVECs were initially seeded into 96-well tissue culture plates. Following the indicated treatments, cell viability was measured by using the Cell Counting Kit-8 (CCK-8, Dojindo Laboratories, Kumamoto, Japan). The optical density (OD) values of CCK-8 were tested at 550 nm.

### Caspase-3 activity

At 30, 000 cells/cm^2^ HUVECs were initially seeded into six-well tissue culture plates. Following the applied HG stimulation, 25 μg of achieved total cell lysates were incubated with DEVD-AFC (the caspase-3 substrate, Invitrogen Thermo-Fisher, Shanghai, China). The relative caspase-3 activity, reflected by the AFC absorbance, was examined through a Fluoroskan Ascent FL equipment machine at 355 nm excitation and 525 nm emission [8].

### Annexin V FACS

HUVECs with the applied treatments were co-incubated with 5 μg/mL of Annexin V and 5 μg/mL of propidium iodide (PI) for 20 min. A fluorescent-activated cell sorting (FACS) equipment (BD bioscience, Shanghai, China) was utilized to sort the apoptotic cells with positive Annexin V staining. Annexin V ratios were recorded.

### TUNEL staining

At 30, 000 cells/cm^2^ HUVECs were initially seeded into six-well tissue culture plates. After the applied HG stimulation, the nuclei were stained with TUNEL (5 μM, Sigma) and DAPI (1 μM, Sigma) for 30 min under the dark. TUNEL ratio (TUNEL/DAPI×100%) was recorded by counting 500 cells from five random views (1 × 200 magnification) from each condition.

### Mitochondrial depolarization

After the applied treatment, mitochondrial depolarization (“∆Ψ”) in HUVECs was measured by a mito-dye JC-1, which shall form green monomers with mitochondrial depolarization in stressed cells [46]. The protocol of JC-1 assay has been described elsewhere [47]. JC-1 green fluorescence absorbance was recorded at the test wavelength of 550 nm.

### Superoxide assay

At 30, 000 cells/cm^2^ HUVECs were initially seeded into six-well tissue culture plates. Following the applied HG stimulation, superoxide contents were tested through a superoxide colorimetric assay kit (BioVision, San Francisco, CA) following the attached protocols. At the wavelength of 450 nm the absorbance of superoxide was tested.

### Glutathione contents

At 30, 000 cells/cm^2^ HUVECs were initially seeded into six-well tissue culture plates. Following the applied HG stimulation, levels of reduced glutathione (GSH) and oxidized disulfide form glutathione (GSSG) were tested [48], using a previously-described protocol [48]. GSH/GSSG ratios were always calculated.

### shRNA

The Nrf2 short hairpin RNA (shRNA) lentiviral particles (sc-37030-V, Santa Cruz Biotech) or the Keap1 shRNA lentiviral particles (sc-43878-V, Santa Cruz Biotech) were individually added to cultured HUVECs (at 60% confluence) for 36h. Stable cells were established by culturing cells in puromycin (5.0 μg/mL)-containing complete medium for 10-12 days. In the stable cells over 95% knockdown of target protein (Nrf2 or Keap1) was achieved. Control cells were transduced with scramble non-sense control shRNA lentiviral particles (“sh-C”).

### Nrf2 knockout

The monoclonal stable HUVECs with the lenti-CRISPR-GFP-Nrf2 knockout (KO) construct, or the Nrf2 KO HUVECs, as well as control cells with an empty vector, were provided by Dr. Xu [8]. Nrf2 KO was always verified by Western blotting and qPCR analyses.

### Statistics analyses

Data were expressed as mean ± standard deviation (SD). Statistical analyses were performed by repeated-measures analysis of variance (RMANOVA) with Dunnett’s post hoc test using a SPSS software (version 21.0, SPSS Inc., Chicago, IL). A two-tailed unpaired T test (Excel 2013, Microsoft) was carried out to examine significance between two treatment groups. ***P*** < 0.05 was considered statistically significant.
